# 

**Published:** 1895-02-02

**Authors:** 


CONTENTS.

**A Teaching University: A New Hope for London Medicine** - 305

**The Lettsomian Lectures** - 306

**Modern Medico-Psychology and Psychiatry.** By T. S. CLOUSTON, M.D. - 307

**Modern Sociology.**—The Case for the Eight Hours Day - 308

**Medical Progress and Hospital Clinics:**

Malignant Disease of the Spine - 311

PROGRESS IN MEDICINE.—Diseases of Children (*continued*) - 311

PROGRESS IN SURGERY.—General Surgery - 312

PROGRESS IN OPHTHALMOLOGY - 314

NEW DRUGS AND PREPARATIONS - 316

NEW APPLIANCES AND THINGS MEDICAL - 316

**Annotations.**—Flat Men—The Memory of Lord Randolph Churchill—Drink Without Drunkenness - 309

**Notes and News** - 310

**Letters from India** - 316

**The Institutional Workshop:**

HOSPITAL ADMINISTRATION - 317

THE STORY OF THE INSANE FROM YEAR TO YEAR - 317

HOSPITAL FESTIVALS AND MEETINGS - 318

HOSPITAL CONSTRUCTION.—The Alice Memorial and the Nethersole Hospitals, Hong Kong - 319

AROUND THE HOSPITALS - 320

**Scraps and Gleanings** - 320

**The Book World of Medicine and Science** - 321

EXTRA SUPPLEMENT.—THE NURSING MIRROR.

**News from the Nursing World.**—Princess Beatrice at Ryde—St. Thomas's Hospital—At the New Hospital for Women—Favoured Fever Nurses—Another Nurses' Home—Helps or Hindrances?—Deaconesses at Tottenham—Faithful Services Acknowledged—Wanted: a Night Nurse—District Nurses at Perth—Incompetent Caretakers—Sick Babies at Florence—Training at Montreal—Short Items - cxxxv

**The Humanising of the Poor Law** - cxxxviii

**Minor Appointments** - cxxxviii

**Elementary Anatomy and Surgery for Nurses.** By W. MCADAM ECCLES, M.B., B.S., F.R.C.S. IV. The Osseus System (*continued*) - cxxxvii

**Notes from Germany.**—Homes for Consumption - cxxxix

**The Book World for Women and Nurses** - cxxxix

**Notes from St. Helena.**—IV. Mistaken Economy - cxl

**Everybody's Opinion** - cxl

**The Hostel of God** - cxl

**Notes and Queries** - cxl

